# General practitioner-centred paediatric primary care reduces risk of hospitalisation for mental disorders in children and adolescents with ADHD: findings from a retrospective cohort study

**DOI:** 10.1080/13814788.2022.2082409

**Published:** 2022-06-17

**Authors:** Angelina Mueller, Olga A. Sawicki, Moritz Philipp Günther, Anastasiya Glushan, Claudia Witte, Renate Klaaßen-Mielke, Ferdinand M. Gerlach, Martin Beyer, Kateryna Karimova

**Affiliations:** a Institute of General Practice, Goethe University, Frankfurt, Germany; b Department of Consultation-Liaison-Psychiatry and Psychosomatic Medicine, University Hospital Zurich, University of Zurich, Zurich, Switzerland; c aQua, Institute for Applied Quality Improvement and Research in Health Care, Goettingen, Germany; d Department of Medical Informatics, Biometry and Epidemiology, Ruhr-University, Bochum, Germany

**Keywords:** ADHD, primary care, multivariate analysis, claims data, health-care-utilisation

## Abstract

**Background:**

General practitioners (GPs) play an essential role in the sustainable management of attention-deficit/hyperactivity disorder (ADHD). To our knowledge, the healthcare programme described here is the first integrated care programme for paediatric ambulatory care embedded in GP-centred-healthcare in Germany.

**Objectives:**

To compare the health-service-utilisation of patients with ADHD enrolled in a GP-centred-paediatric-primary-care-programme with usual care in terms of disease-related hospitalisation, pharmacotherapy and psychotherapy.

**Methods:**

In 2018, we conducted a retrospective cohort study of 3- to 18-year-old patients with ADHD in Baden-Wuerttemberg, southern Germany. The intervention group (IG) comprised patients enrolled in a GP-centred-paediatric-primary-healthcare-programme and consulted a participating GP for ADHD at least once. GP-centred-paediatric-primary-care provides high continuity of care, facilitated access to specialist care, extended routine examinations and enhanced transition to adult healthcare. Patients in the control group (CG) received usual care, meaning they consulted a non-participating GP for ADHD at least once. Main outcomes were disease-related hospitalisation, pharmacotherapy and psychotherapy. Multivariable logistic regression was performed to compare groups.

**Results:**

A total of 2317 patients were included in IG and 4177 patients in CG. Mean age was 8.9 ± 4.4. The risk of mental-disorder-related hospitalisations was lower in IG than CG (odds ratio (OR): 0.666, 95% confidence interval (CI): 0.509–0.871). The prescription rate for stimulants was lower in IG (OR: 0.817; 95% CI: 0.732–0.912). There was no statistically significant difference in the participation rate of patients in cognitive behavioural therapy between groups (OR: 0.752; 95% CI: 0.523–1.080).

**Conclusion:**

Children and adolescents with ADHD enrolled in GP-centred-paediatric-primary-care are at lower risk of mental-disorder-related hospitalisation and less likely to receive stimulants.

KEY MESSAGES

- We evaluated the first GP-centred paediatric primary care programme in Germany for 6494 children and adolescents with ADHD.
- Children and adolescents with ADHD enrolled in strong paediatric primary care are at lower risk of mental disorder-related hospitalisation.
- The prescription rate for stimulants was lower in the intervention group. There was no statistically significant difference in the participation rate of patients in cognitive behavioural therapy between groups.

## Introduction

Primary care plays a substantial role in managing mental disorders in minors [1]. It is unclear who should be responsible for delivering care to patients with ADHD but a stepped approach would appear to be the most suitable strategy [2]. NICE 2018 guidelines consider continuity of care to be the most crucial factor in managing ADHD. This means general practitioners (GPs) should take on a central role for patients and family members and collaborate with paediatric and psychiatric colleagues where necessary. Moreover, guidelines note that collaborative models have strengthened the role of GPs in pharmacological treatment of ADHD [3]. However, GPs participate in differing patient care programmes; to our knowledge, there is little research on their effectiveness.

We considered the number of hospitalisations for mental disorders, prescriptions of psychopharmaceuticals, and the need for psychotherapy as possible for comparison between usual care and novel models.

### GP-centred paediatric primary care

The paediatric care programme described in this study is part of the GP-centred care programme by AOK health insurance in Baden-Wurttemberg, Germany, focusing on children and adolescents. The benefits and components of GP-centred care have been described elsewhere [4,5]. Further details and the legal framework can be found in the German Social Code, Book 5 (SGB V) §73c [6]. In GP-centred paediatric primary care, patients receive care from a single GP responsible for coordinating care and referring them to specialists. In contrast to treatment, as usual, GP-centred paediatric primary care includes extended preventive paediatric check-ups and innovative services such as advanced screening for diseases in children and adolescents, as well as hearing and vision tests (extended amblyopia screening). In addition, financial incentives are provided to encourage GPs to extend consultation times for young patients with psychosocial problems. We analysed in our study whether these differences in coordination of care affected prescriptions of psychopharmaceuticals and the need for psychotherapy when comparing usual care and novel models.

Furthermore, waiting times for specialist appointments were reduced compared to usual care. GPs and paediatricians participate in the programme, whereby enrolment is voluntary for patients and doctors. Regular care also involves contacting GPs and paediatricians but there are no official obligatory further requirements except for routine check-ups.

In both care models, it is not only GPs and paediatricians involved in the therapeutic process but also other specialists (e.g. psychologists, psychiatrists, neurologists, child psychiatrists and psychotherapists). ADHD is diagnosed according to World Health Organisation recommendations and German guidelines for ADHD treatment [6,7]. In summary, this requires attention deficit (for AD disorder) and/or hyperactivity and impulsivity (for ADHD or hyperactivity disorder) with symptoms present for longer than six months, start of symptoms before age seven, significant impairment of functioning or suffering in more than one context (i.e. in school and at home), and no other mental disorders to adequately explain these symptoms (i.e. no autism, affective or anxiety disorder) [7].

Patient empowerment and shared decision making are recommended components of guideline compliant therapy [8] in both treatment settings. Cognitive behavioural therapy and psychopharmacological treatment with stimulants are considered the cornerstones of evidence-based treatment of ADHD. However, as some symptoms of ADHD may result from other factors, careful exploration of patients and family circumstances is necessary to avoid jumping to erroneous conclusions prematurely [9]. Data on the treatment of ADHD in primary care settings in Germany is scarce. Apart from studies based on local data [10], little is known about health care utilisation in this patient group in (southern) Germany.

The present study aims to assess whether the GP-centred paediatric primary care programme effectively reduces psychiatric hospitalisation rates, prescriptions of psychopharmaceuticals, and the need for psychotherapy.

## Methods

### Study population and setting

We conducted a retrospective cohort study based on claims data from 1,555,707 patients with mental health disorders, of whom 193,296 were 18 years old or younger. Claims data were provided by the statutory health insurance fund ‘Allgemeine Ortskrankenkasse’ (AOK) in Baden-Wuerttemberg, southern Germany, for 2016 to 2018. Baden-Wuerttemberg had about 11.1 million inhabitants [11], of whom 5.1 million were insured by AOK (about 1.9 million underages) in the study period. AOK is the largest health insurance fund in this federal state, covering about 80% of the insured population [12]. In 2018, about 5000 doctors and 1.6 million patients were enrolled in GP-centred care. Overall, 383 doctors (paediatricians and GPs in private practice) and 168,901 patients were enrolled in GP-centred paediatric primary care in 2018.

### Patient data

Inclusion criteria for patients were: diagnosis of ADHD (ICD-Code: F90.0–F90.9) in 2017, continuous insurance status, residence in Baden-Wuerttemberg, and aged 18 years or younger.

**Figure 1.**
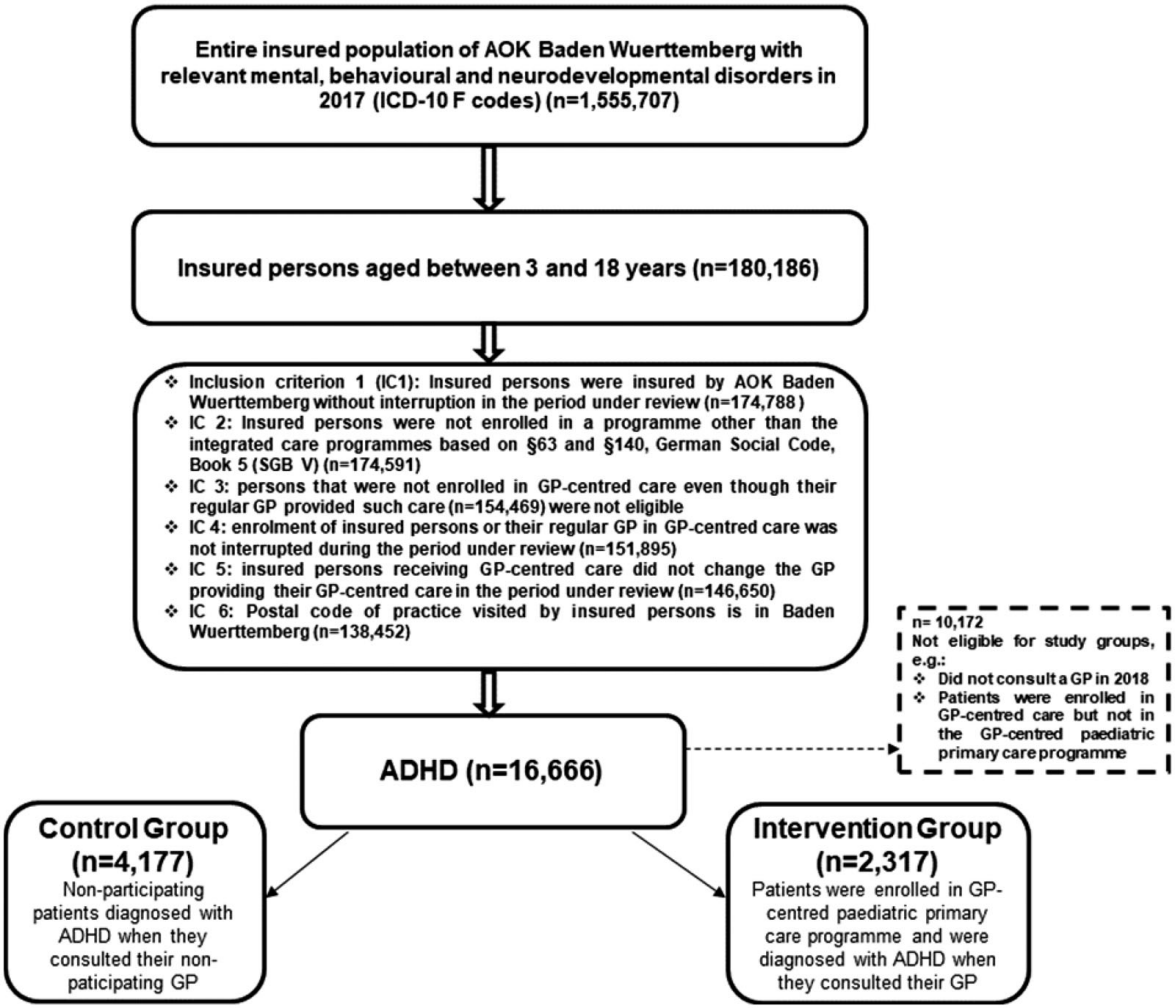
Study population with inclusion criteria.

Patients under three years of age were later excluded. Inclusion criteria are shown in Figure 1. The follow-up period ended in 2018. Patients’ comorbidities and baseline characteristics (i.e. sex, congenital malformation, allergies, asthma, obesity and ADHD diagnosis) were recorded in 2017, and the relevant Charlson comorbidity index, age and nursing level (i.e. medical level of care, reaching from 1 to 5 for children) in 2018. The intervention group comprised patients enrolled in the GP-centred paediatric care programme that had consulted a GP enrolled in the paediatric care programme when ADHD was first diagnosed. When a specialist or another health care provider (not a GP) was responsible for the initial diagnosis of ADHD, the first GP contact following that diagnosis was assessed. The control group consisted of patients not enrolled in either the GP-centred paediatric care or the GP-centred care programme and had consulted a GP that did not participate in the GP-centred care programme at least once.

The patients or their legal representatives had to provide their written informed consent before enrolment in the programme. Ethical approval was obtained from the local ethics committee of the Department of Medicine of Goethe-University in Frankfurt (No. 470/13).

Articles on this study were prepared following the STROBE Statement and the German reporting standard for secondary data analysis (STROSA) [13]. The study is part of an extensive evaluation report on GP-centred care in Germany (HZV-Broschüre [14]).

### Quality of data

The quality of the claims data used in this study was subject to several quality checks. First, the Association of Statutory Health Insurance Physicians and Pharmacies provided data that had been submitted to a data collection and verification service by physicians provided by AOK health insurance. Secondly, the data was transferred to the aQua data management institute, an independent centre in Goettingen, Germany, where they were subjected to further quality controls and aligned with our definition of variables and indicators. The following quality checks were part of the aQua quality controls performed with SPSS: acceptance and first check of data (data kind, number of data and readability), reading in and reviewing the data tables (file size, etc.), checking of data integrity (especially linkability of Tables), checking the completeness of the data (related to the number of observations in tables, characteristics within tables or also in linked tables, missings, duplications) and plausibility check (related to frequencies of characteristic expressions as well as to simple distinct values like mean, min, max, SD etc.) The expressions are then also compared with those from previous years. The data manager in our study team also performed a plausibility check.

### Outcomes

The primary outcome in this study was hospitalisation for a mental disorder (ICD-10-F-code) on at least one occasion in 2018. Hospitalisation due to a mental disorder (=disease-related hospitalisation) was defined as the use of an in-hospital medical service by a patient diagnosed with a mental disorder. Further clinical outcomes included the prescription of a psychopharmaceutical on at least one occasion and psychotherapy in 2018. ATC codes used for psychopharmaceuticals were those for stimulants (N06BA12, N06BA21, N06BA09, N06BA01, N06BA02, N06BA03, N06BA04). All measures were based on administrative data. Other results were assessed as part of the evaluation study.

#### Statistical analysis

##### Descriptive statistics

Initially, all target and influencing variables were analysed descriptively. The number of non-missing values and values for the mean, standard deviation, median, first and third quartile, minimum and maximum were specified for continuous variables, and absolute and relative frequencies calculated for categorical variables.

##### Regression model

Dichotomous variables were evaluated using the multivariable regression model. Depending on the outcome variable, either logistic or negative-binomial regression models were used. Results were presented as odds ratios (OR) for binary variables and rate ratios (RR) for count variables, with 95% confidence intervals. We considered two-sided *p*-values and labelled *p*-values <0.05 as statistically significant.

Covariables were defined based on literature and prior evaluation studies. The model used the following covariables: age, sex, nursing level, congenital malformations, allergies, asthma, obesity, Charlson comorbidity index and rural area of residence. Baseline characteristics are displayed in Table 1.

**Table 1.** Baseline characteristics of study groups.

Baseline characteristics	Control group (*n* = 4177)	Intervention group (*n* = 2317)	*p*-Value^a^
Age (all) mean [SD]	12.1 ± 3.4	11.9 ± 2.9	0.010
Sex (female)	23.9%	26.2%	0.043
Charlson Comorbidity Index mean [SD]	0.2 ± 0.5	0.1 ± 0.4	0.018
Nursing level	6.8%	5.0%	0.004
Congenital malformation	9.1%	9.%	0.808
Allergies	3.6%	3.6%	0.904
Asthma	9.4%	10.7%	0.100
Obesity	6.2%	8.4%	<0.001
Urban area	49.2%	52.8%	0.005
Diagnosed with ADHD in 2017	92.7%	93.8%	0.073

^a^*t*-Test for count and continuous variables, chi-square test for binary variables.

All descriptive and comparative analyses were carried out using SAS (version 9.4) and IBM SPSS Statistics (version 25).

## Results

Overall, 6494 cases met our inclusion criteria: patients aged 3–18 years diagnosed with ADHD. We included 4177 insurants in the control group and 2317 in the intervention group, respectively. Results are shown in Table 2.

**Table 2.** Descriptive statistics and results of the multivariable analysis.

Outcome	Control group *n* = 4177	Intervention group *n* = 2317	OR^a^	RR	95%-confidence interval		*p*-Value
Disease-related hospitalisation [%]	5.0%	3.4 %	0.666	N.A	0.509	0.871	0.003
prescribed stimulants [%]	38.5%	34.5 %	0.817	N.A	0.732	0.912	0.0003
cognitive behavioural therapy [%]	10.2%	7.8 %	0.752	N.A	0.523	1.080	0.122
number of uncoordinated specialist visits (quarterly, maximum 4) mean [SD]	*n* = 1106 2.46 ± 1.36	*n* = 586 2.25 ± 1.46	N.A	0.914	0.855	0.976	0.0076
GP visits (quarterly, maximum 4) mean [SD]	3.02 ± 1.05	3.23 ± 0.87	N.A	1.073	1.042	1.104	<0.0001

^a^For count variables we estimated the rate ratio (RR), for binary variables the odds ratio (OR). The presented OR and CI are adjusted results.

### Disease-related hospitalisation

Before adjustment for relevant comorbidities, disease (mental health disorder)-related hospitalisation was lower in the intervention group (3.4% vs. 5.0%). After adjustment, differences between groups remained significant and the chance of hospitalisation continued to be lower in the intervention group (OR 0.666; 95% CI 0.509–0.871; *p* = 0.003).

### Psychostimulant and psychotherapy prescription rates

Prescription rates of stimulants to minors were lower in the intervention group than in the control group (38.5% vs. 34.5%). Multivariable logistic regression revealed that assignment to the intervention group was associated with fewer stimulant prescriptions per patient (OR: 0.817; 95% CI: 0.732–0.912; *p* = 0.0003)

Descriptive analysis indicated a lower rate of cognitive behavioural therapy in the intervention group (7.8% vs. 10.2%) but the difference between groups was not significant (OR 0.752; 95% CI: 0.523-1.080; *p* = 0.122).

### Continuity and coordination of care

Children and adolescents in GP-centred paediatric care were more likely to receive coordinated care, and uncoordinated visits to specialists without GP referrals were lower (RR: 0.914; 95% CI: 0.855–0.976; *p* = 0.0076). Additionally, the risk of consulting a GP was higher in the intervention group (RR: 1.073; 95% CI: 1.042–1.104; *p* < 0.0001).

## Discussion

### Main findings

In this retrospective cohort study of 6494 ADHD patients aged 3–18 years old (female 24.7%), we found that children and adolescents with ADHD enrolled in GP-centred-paediatric-primary care are at lower risk of mental-disorder-related hospitalisation and less likely to receive stimulants.

Multivariable logistic regression analysis of administrative data for children and adolescents with ADHD revealed that participation in GP-centred paediatric primary care reduced the risk of mental health disorder-related hospitalisation. Furthermore, patients in the intervention group had a lower stimulant prescription rate while there was no statistically significant difference in the participation rate of patients in cognitive behavioural therapy between groups.

### Interpretation

We chose to include children aged three years and older to comply with German guidelines for ADHD [15]. In the United States, studies describe greater prevalence of ADHD in boys than girls (147 vs. 62 per 1000), with a stimulant prescription rate of about 80% per visit [16]. Another study reports up to 90.1% [17]. Our results are consistent in terms of higher distribution of ADHD in males. The Anatomical Therapeutic Chemical codes used in this study are the same as in the previous cohort study [18].

Our results show lower rates of stimulant prescriptions in patients enrolled in the GP-centred paediatric primary care programme. This is important because other studies have shown a trend towards increased use of prescription stimulants in recent years [18] – possibly in conjunction with over-diagnosis of ADHD [9]. Unfortunately, we cannot claim whether prescription of stimulants was necessary in the groups or whether patients, in fact, required a higher amount of prescriptions since we do not know the clinical circumstances. Similarly, most recent research has shown that 9-year-old children treated for ADHD have more emotional and peer relationship problems, worse prosocial behaviour, and poorer self-concept [19]. Careful evaluation of the need for stimulant prescriptions in minors with symptoms of hyperactivity and inattention may require more time than is available to GPs in standard treatment settings. Therefore, the difference in time available for therapy could be an essential reason for differences in prescription rates.

Recent research indicates that an intervention addressing both individual staff and organisational factors may effectively improve the implementation and quality of mental health services in paediatric primary care [20]. Another important feature of the intensified treatment programme described here is the structured transition from paediatric to adult healthcare. ADHD patients could particularly benefit from such healthcare continuity [2].

Psychotherapy can help patients with ADHD, whether or not a patient is taking stimulants [21]. We should consider that we might not have measured all the psychotherapy interventions the patients received since we only included those accepted by the health insurance. Lack of resources available for these interventions seems to be a known problem and could be one explanation for only 8–10% of patients in both groups receiving psychotherapy [22].

Although we primarily considered patients consulting a GP, it is possible that further care was provided by specialists and/or paediatricians. Positive effects such as lower risk of disease-related hospitalisation may reflect both the intensified treatment programme and appropriate collaboration with specialists [23].

### Strengths and limitations

Major strengths of the study are the high number of eligible patients and cross-sectoral (inpatient and outpatient) real-world data. In Germany, we found only a few studies based on claims data that dealt with mental disorders in minors, especially in outpatient care.

In our study, we used an advanced model for comorbidity adjustment. Nonetheless, we cannot rule out residual confounding, as claims data do not reveal all clinical confounders.

One limitation of the study is that studies conducted in the 1990s showed that children receiving methylphenidate were more likely to use health services, e.g. emergency care [24]. Thus, lower use of prescription stimulants may lead to a decline in hospitalisation rates and *vice versa*.

Similar studies have previously described common limitations associated with the use of claims data [5,25]. Most importantly, clinical data and information on disease intensity are not available in secondary data. We want to point out that GP-centred paediatric primary care does not focus on ADHD or mental disorders in general, but is a programme that is accessible to all patients aged under 18 and enrolled in GP-centred care. Since enrolment in GP-centred care is voluntary for both physicians and patients, self-selection bias should be considered during data interpretation. It is, for example, possible that the patient cohort in the intervention group showed greater treatment adherence. We were also unable to evaluate the consultation and the quality of care in detail, so the described effects are probably multifactorial. Patients in the intervention group certainly profited from high continuity of care and more contact with their GP [26]. Nonetheless, it is possible that consulting different specialists impacted patient outcomes. Most of the eligible patients were diagnosed with ADHD, yet in 2017 (Table 1) we have no further information on the duration of disease or whether inpatient care was previously necessary.

## Conclusion

GP-centred paediatric primary care is associated with a lower risk of hospitalisation. Children and adolescents with ADHD enrolled in GP-centred-paediatric-primary-care are less likely to receive stimulants.

### Implications

Our results possibly indicate the need for therapeutic strategies in outpatient care. Since a lack of education in ADHD has been identified as a significant problem [27], implementing educational programmes may be beneficial in this setting. Process evaluation may also provide further insights and enable the influence of individual factors in strengthening outpatient care to be identified.

Qualitative research on the quality of care in children and adolescents with ADHD in a primary care setting in Germany is recommended.
